# A Case Report of Bezoar-Induced Small Bowel Obstruction: A Potential Surgical Complication of Semaglutide

**DOI:** 10.7759/cureus.99664

**Published:** 2025-12-19

**Authors:** Cambo Keng, Sze Mun Thor, Ramana Balasubramaniam, Frans Pretorius

**Affiliations:** 1 General Surgery, Goulburn Valley Health, Shepparton, AUS

**Keywords:** bezoar, gastroparesis, glp-1 receptor agonist, semaglutide, small bowel obstruction, small bowel resection

## Abstract

Bezoars are an uncommon but recognised cause of small bowel obstruction (SBO) and are difficult to differentiate preoperatively from other aetiologies. We report a rare case of acute SBO due to a bezoar in a 65-year-old woman who had been on semaglutide, a Glucagon-like peptide-1 (GLP-1) receptor agonist therapy for weight loss. She presented with a four-day history of abdominal pain, distension, nausea, and vomiting. Imaging confirmed SBO. Following unsuccessful nonoperative management, she underwent diagnostic laparoscopy and laparoscopic resection of a jejunal segment containing a large phytobezoar. We postulated that semaglutide-induced gastroparesis and slow intestinal transit may have contributed to bezoar formation and subsequent bowel obstruction. This case highlights a rare but important surgical adverse effect, particularly in patients receiving GLP-1 receptor agonists who present with bowel obstruction.

## Introduction

Bezoar-induced small bowel obstruction (SBO) is an uncommon but recognised cause of intestinal obstruction, accounting for 0.4% to 4% of cases [1]. Bezoars are intraluminal concretions formed from undigested material, most often originating in the stomach before migrating into the small intestine [2,3]. Among the small bowel segments, the terminal ileum is the most frequent site of obstruction due to its relatively narrow lumen and slower motility [1,3]. Bezoars are typically classified into four groups: phytobezoars (plant material), trichobezoars (hair), lactobezoars (milk proteins), and pharmacobezoars (medications), with phytobezoars being the most common [4]. Established risk factors include high-fibre diets, prior gastric surgery, and medical conditions or medications that impair gastrointestinal transit [3,5].

Glucagon-like peptide-1 receptor agonists (GLP-1RAs), such as semaglutide, are increasingly used for type 2 diabetes and weight management [6]. At therapeutic doses, these agents slow gastric emptying through coordinated effects on antral contractility, proximal gastric relaxation, and increased pyloric tone [7]. Emerging clinical observations suggest a possible association between GLP-1RA use and gastric retention or bezoar formation [8,9], although this relationship remains incompletely defined.

We present a rare case of SBO secondary to a phytobezoar in a patient receiving semaglutide therapy. This case highlights a potential association that warrants clinical awareness, particularly as GLP-1RAs become more widely used across diabetic and non-diabetic populations.

## Case presentation

A 65-year-old female patient presented to the emergency department after an episode of large watery stool, followed by a four-day history of progressively worsening epigastric and central abdominal pain, associated with nausea, vomiting, abdominal distension, and obstipation. She reported no urinary, gynaecological, infective, or constitutional symptoms. She denied sick contacts or unusual dietary intake, adhering to her usual diet of salad and bread.

This occurred on the background of a laparoscopic hysterectomy 15 years prior. Her other past medical history included hypertension, hypercholesterolaemia, ischaemic heart disease with prior coronary stenting, and rheumatoid arthritis. She had no history of diabetes mellitus or previous gastric surgery. She had been receiving semaglutide 0.5 mg weekly for approximately six months, with no recent dose changes. Her regular medications included aspirin, rosuvastatin, candesartan, sertraline, and sulfasalazine.

On examination, she was haemodynamically stable. The abdomen was distended and tender without guarding or peritonism. Digital rectal examination was unremarkable. Laboratory investigations showed a haemoglobin of 154 g/L, white cell count of 12 × 10⁹/L, C-reactive protein of 93 mg/L, lipase of 18 U/L, and troponin of 8 ng/L. Liver and renal function tests were within normal limits.

**Figure 1:**
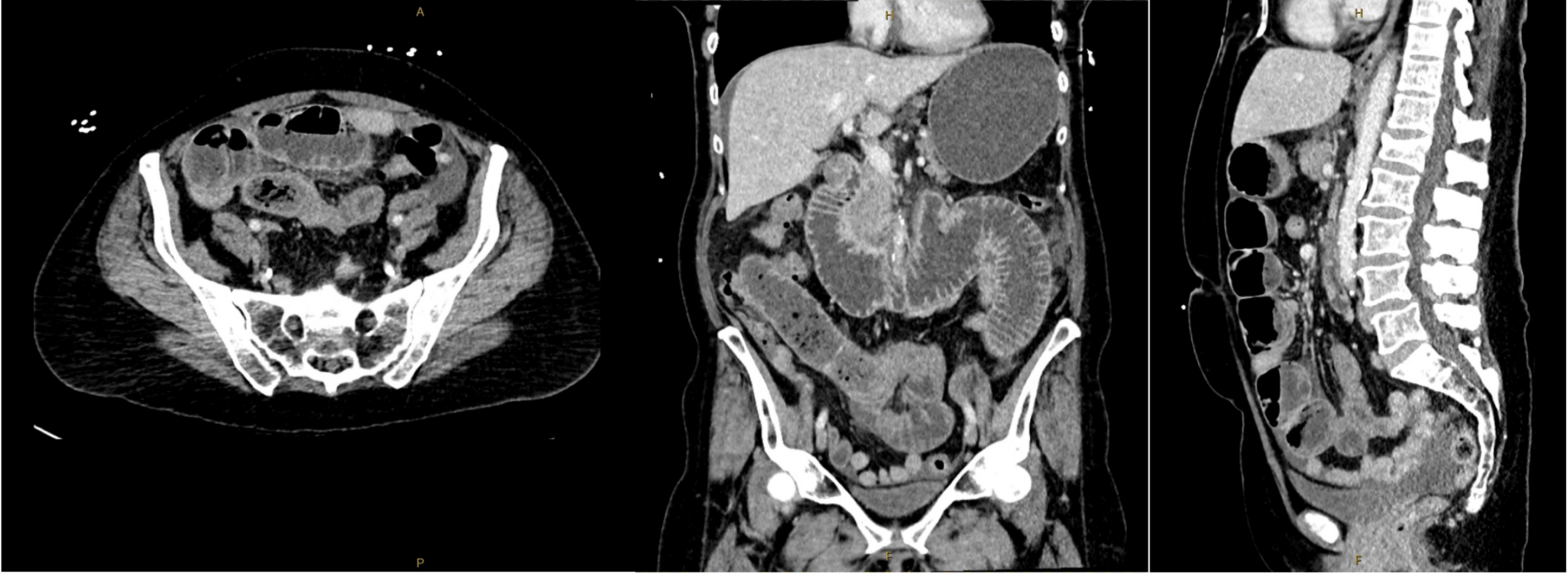
Contrast-enhanced CT scan of the abdomen and pelvis demonstrating dilated small bowel loops with multiple air-fluid levels and a transition point in the low central abdomen. No discrete intraluminal mass or volvulus were identified

Contrast-enhanced computed tomography (CT) of the abdomen and pelvis demonstrated dilated small bowel loops with multiple air-fluid levels and a transition point in the low central abdomen, associated with mesenteric venous congestion (Figure 1). No discrete intraluminal mass or mesenteric twist was identified. A small volume of free fluid was noted, without evidence of free gas or bowel ischaemia.

Given her prior surgical history, the patient was presumed to have an adhesional SBO and was therefore managed conservatively with nil-per-oral status, nasogastric decompression, intravenous fluids, analgesia, electrolyte correction, and Gastrografin follow-through. Unfortunately, she failed the gastrografin challenge due to persistent nausea and vomiting, and a decision was made to proceed with operative intervention.

**Figure 2:**
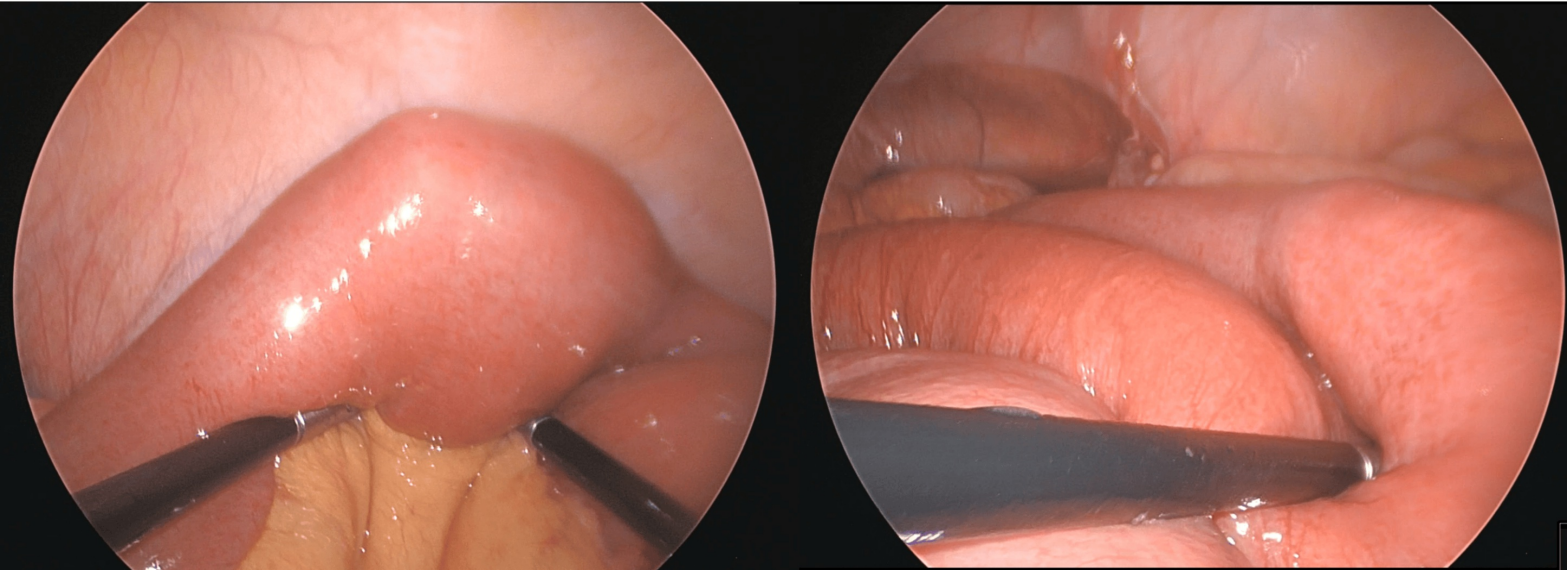
Intraoperative laparoscopic images showing a transition point in the proximal jejunum with a large obstructive intraluminal mass and upstream bowel dilatation

**Figure 3:**
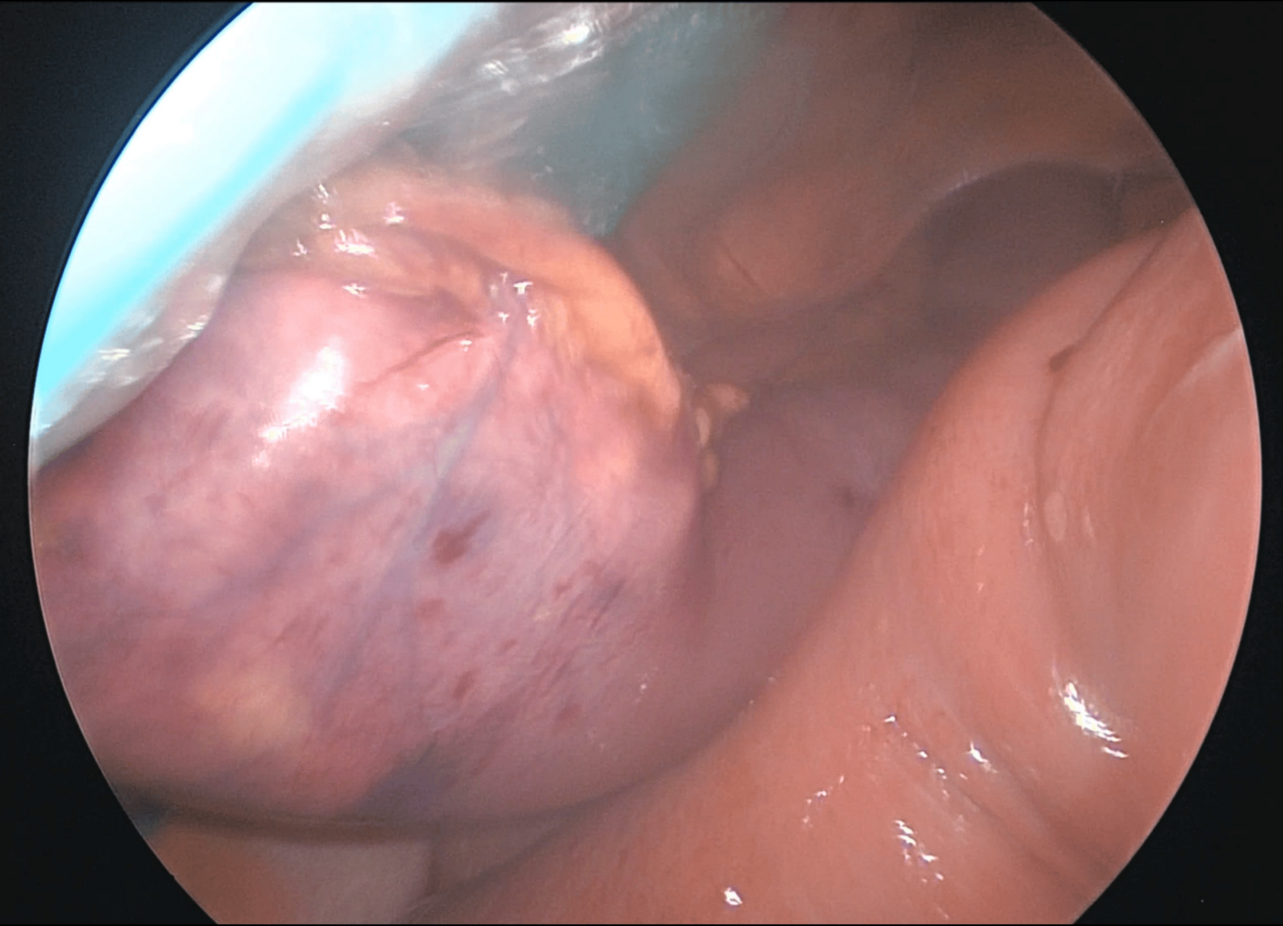
Intraoperative laparoscopic image demonstrating resected segment of jejunum containing bezoar being extracted through the abdominal retractor

**Figure 4:**
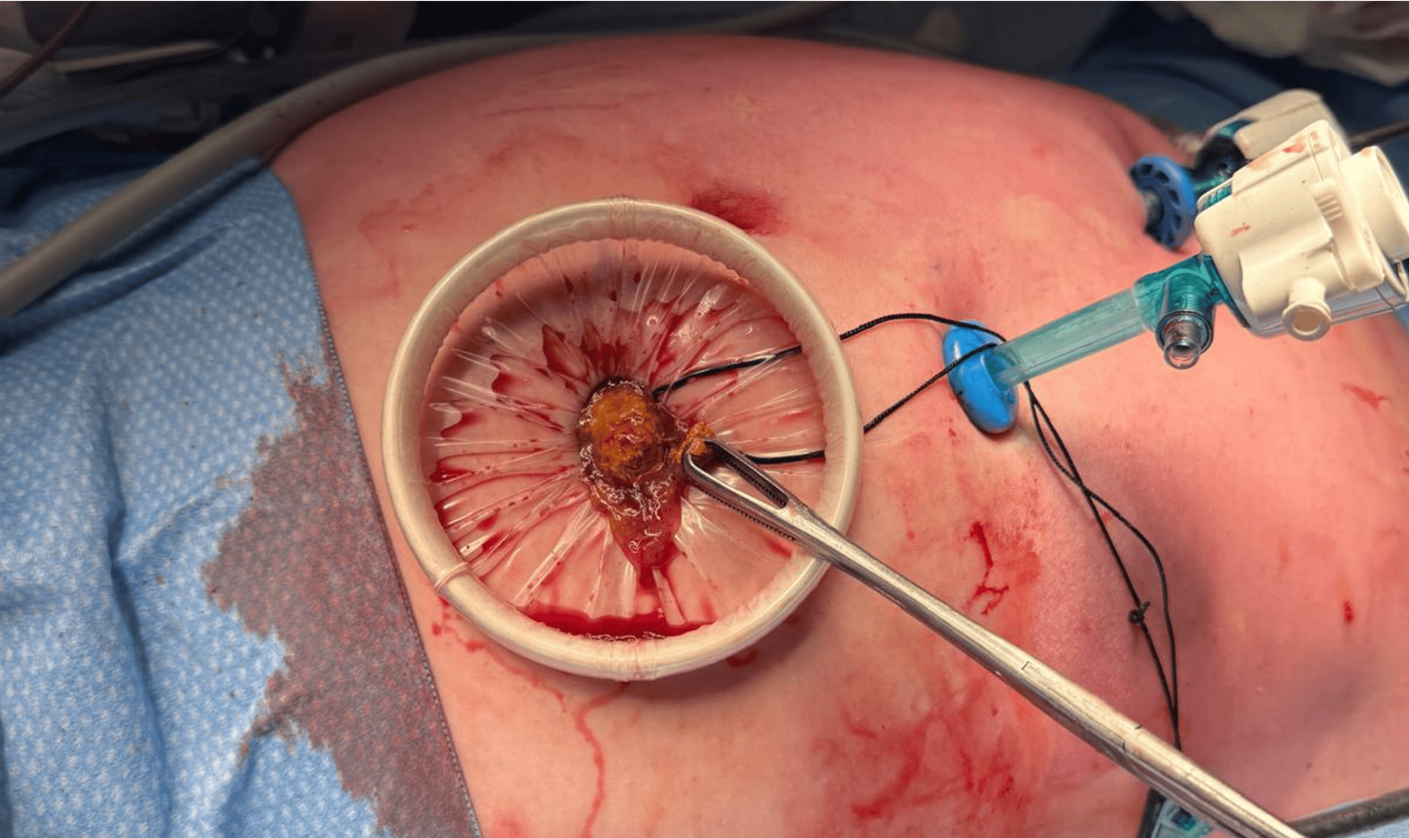
Intraoperative photograph demonstrating retrieval of the resected jejunal segment containing a phytobezoar through a laparoscopic port site. The bezoar is visible protruding through the Alexis retractor during specimen extraction

Exploratory laparoscopy revealed a markedly distended stomach, duodenum, and proximal jejunum. Furthermore, a firm adherent intraluminal mass was identified in the proximal jejunum, concerning for an obstructing small bowel tumour (Figures 2-3). A 30 cm segment of jejunum was resected, followed by a stapled side-to-side anastomosis. No other adhesions or masses were identified. The resected specimen ruptured intraoperatively during retrieval, revealing a firm mass composed of plant fibres, suggestive of a phytobezoar (Figure 4). Histopathology confirmed mucosal ischaemia with patchy necrosis, without evidence of malignancy, vasculitis, or thromboembolism.

Postoperatively, the patient had a slow but uncomplicated recovery. She was referred to a dietitian and advised to commence a low-fibre diet. A post-operative CT enterography showed no small bowel lesion, stricture, or residual bezoar. She remained clinically well at the outpatient review.

## Discussion

To our knowledge, this is among the first reported cases of bezoar-induced SBO in patients receiving GLP-1RA therapy, highlighting a potentially under-recognised surgical complication. The intraoperative finding of a bezoar prompted reconsideration of underlying risk factors.

Predisposing factors for bezoar formation include high-fibre diets, systemic conditions impairing gastrointestinal motility, and prior gastric surgery [1,3]. Relevant systemic conditions include diabetes mellitus, hypothyroidism, inflammatory bowel disease, malignancies, neurological disorders, and medications affecting gastrointestinal transit [4,10]. The patient had no such risk factors apart from being on semaglutide therapy, a long-acting GLP-1RA, for obesity treatment.

Gastrointestinal symptoms such as nausea, vomiting, constipation, diarrhoea, abdominal pain, and distension are some of the most common adverse effects of semaglutide and are usually the reason for discontinuation of treatment [11]. GLP-1RAs are known to delay gastric emptying by altering antral motility, increasing pyloric tone, and promoting proximal gastric relaxation [7]. Recent literature has described retained gastric content and gastric bezoars detected during esophagogastroduodenoscopy in patients treated with GLP-1RAs [8,9]. Furthermore, GLP-1 and GLP-1RA are associated with the inhibition of migrating motor complex and increased small bowel transit time in patients with and without diabetes [12]. The specific mechanism for inhibition of intestinal motility is yet to be fully elucidated; however, it is thought to occur via vagal and direct central nervous system effects [12]. The delayed gastric emptying and impaired intestinal transit associated with GLP-1RA therapy likely played a causal role in bezoar formation in our patient.

CT imaging remains the first-line diagnostic modality for SBO, although bezoars may be difficult to detect [13]. When visualised, bezoars appear as well-defined, mottled, intraluminal masses [1]. In our case, the bezoar was not appreciated on preoperative imaging, underscoring the diagnostic challenges and importance of maintaining clinical suspicion, particularly in patients on agents known to impair gastric motility. A retrospective study by Gao et al. also suggested that CT features such as bezoar morphology, bowel wall thickening, mesenteric haziness, and peritoneal fluid could predict failure of non-operative management [13].

Management of bezoar-induced SBO typically begins conservatively, but surgical intervention is required in cases of clinical deterioration, failed conservative therapy, or complications such as bowel ischaemia [10]. Non-surgical approaches, including enzymatic dissolution or mechanical fragmentation (e.g., with Coca-Cola®), have variable success [5,14]. Surgical options include manual fragmentation, enterotomy with extraction, or segmental resection if necrosis or perforation is present [10,15].

While laparoscopic management, as employed in our case, offers the benefits of minimally invasive surgery, it requires technical expertise and can limit tactile exploration of the bowel, a consideration given that synchronous multiple bezoars occur in up to one-third of cases [15]. As in our case, we were not able to feel the mass and, therefore, were unable to confidently differentiate a bezoar from a small bowel tumour. Furthermore, there is a risk of unrecognised bezoars which may be caught in the staple line and predispose to anastomotic complications [8]. In fact, in patients undergoing bariatric surgery or gastric resection while receiving GLP-1RA therapy, preoperative gastroscopy is recommended [8].

Preventive strategies are central to management, including dietary modification (particularly avoidance of high-fibre foods), ensuring adequate mastication, and considering prokinetic therapy in high-risk patients [1,16]. Current evidence does not support routine discontinuation, dose reduction, or pre-emptive imaging in asymptomatic users; however, emerging reports of retained gastric contents and delayed gastric emptying have led some authors to recommend withholding GLP-1RAs prior to upper gastrointestinal or bariatric procedures, or considering pre-operative endoscopy in selected high-risk patients. In our patient, semaglutide was discontinued postoperatively, given its potential contribution to impaired motility. Ultimately, decisions regarding continuation versus cessation should be individualised, balancing metabolic benefits against gastrointestinal risks.

Given the widespread and growing use of semaglutide among both diabetic and non-diabetic populations, awareness of bezoar-related SBO is crucial for early diagnosis and prompt surgical intervention. Timely surgical intervention and appropriate postoperative counselling are key to reducing morbidity and preventing recurrence. As this is a single case report, a causal relationship cannot be confirmed, and unrecognised patient-specific or dietary factors may have contributed to bezoar formation independent of semaglutide use.

## Conclusions

This case highlights bezoar-induced SBO as a rare but important diagnostic consideration in patients receiving semaglutide. While a plausible association exists through GLP-1RA-related delay in gastric emptying and intestinal transit, causality cannot be confirmed, and other unrecognised factors such as dietary fibre content, adhesions, or baseline variations in motility may also have contributed. Clinicians should maintain awareness of this potential association when evaluating obstructive symptoms in patients on GLP-1RAs, with attention to early diagnosis, timely surgical intervention, and appropriate postoperative counselling.
